# Branched-chain amino acids in skeletal and cardiac muscles: a review of metabolic variations and cancer’s impact

**DOI:** 10.3389/fphys.2026.1943433

**Published:** 2026-10-22

**Authors:** Julian Chrzanowski, Wojciech Graboń, Karolina Buchajska, Agnieszka Cudnoch-Jędrzejewska

**Affiliations:** 1 Chair and Department of Physiology and Pathophysiology, Medical University of Warsaw, Warsaw, Poland; 2 Chair and Department of Biochemistry, Medical University of Warsaw, Warsaw, Poland

**Keywords:** BCAA catabolism, branched-chain amino acids, cancer cachexia, cardiac muscle, muscle wasting, skeletal muscle

## Abstract

Branched-chain amino acids (BCAA) - leucine, isoleucine, and valine - serve as essential regulators of energy homeostasis, protein turnover, and nutrient signaling in striated muscle. However, their metabolic processing varies substantially across tissues. This review contrasts BCAA metabolism in skeletal versus cardiac muscle and evaluates how cancer alters it. Skeletal muscle functions as the main site for whole-body BCAA transamination and oxidation, distributing these amino acids between protein synthesis and oxidative catabolism in line with physiological needs. In comparison, the myocardium maintains low baseline BCAA oxidation, depends primarily on fatty acids and glucose, and tightly limits BCAA catabolic flux to prevent the buildup of intermediates that would undermine mitochondrial function. In malignancies, BCAA metabolism is reconfigured in a way that depends on tumor type: many tumors increase BCAA uptake and suppress catabolism in order to preserve intracellular stores, as a result maintaining mechanistic target of rapamycin complex 1 (mTORC1) signaling and supporting proliferation. Using lung, pancreatic and colorectal cancers as main examples, we outline how this tumor-driven metabolic rewiring is linked to cancer cachexia - a multi-organ syndrome in which inflammation, mitochondrial dysfunction, and impaired anabolic signaling promote muscle and cardiac wasting rather than simple substrate limitation. We describe first the mechanisms shared by skeletal and cardiac muscle, then the features specific to each tumor type, and we distinguish throughout between cardiac evidence obtained in tumor-bearing hosts and inferences extrapolated from heart failure. We conclude that interventions directed at BCAA are most reasonable early in the disease course, and only when paired with approaches that suppress inflammation and restore mitochondrial quality control. We also identify priorities for tissue-specific biomarkers and for future research.

## 1 Introduction

Metabolic pathways of branched-chain amino acids (BCAA) - valine, isoleucine and leucine - are important in maintaining energy homeostasis, protein dynamics and function of skeletal and cardiac muscles. They are not only major sources of amino acids for protein synthesis, but also act as regulators of signal transduction pathways as well, for example influencing mTOR pathway that controls cell growth and metabolism (Lynch and Adams, 2014; Nie et al., 2018). Moreover, BCAA are involved in energy production in mitochondria through catabolism, which is increased during exercise or other forms of metabolic stress (Shimomura et al., 2004). Interestingly, their utilization and functional significance appear to be tissue-dependent.

There are many metabolic similarities between skeletal and cardiac muscles, but there is also an increasing amount of differences being discovered each year. Skeletal muscle is the primary site of BCAA oxidation and it plays an important role in the overall nitrogen balance of the body, especially after physical activity (Mann et al., 2021). On the other hand, cardiac muscle strictly controls its BCAA turnover in order to avoid mitochondrial dysfunction caused by accumulation of toxic BCAA-derived metabolites. It relies more on fatty acid oxidation to function at a high energy demand (Sun et al., 2016). These differences show how different the expression of enzymes - branched-chain aminotransferase (BCAT) and branched-chain α-keto acid dehydrogenase (BCKDH) complex - involved in BCAA catabolism is, depending on the type of muscle involved (Hutson et al., 2005).

Apart from their catabolic fate, BCAA also serve as a fundamental anabolic agent - as important substrates for protein synthesis and growth promotion. In skeletal muscle, BCAA - especially leucine - stimulate mTORC1-dependent translational control, which enhances muscle protein synthesis in response to feeding and exercise, provided that total essential amino acid availability and hormonal context are permissive (Lynch and Adams, 2014; Norton and Layman, 2006; Wolfe, 2017). At the same time, the same logic extends to rapidly proliferating cells: increased BCAA uptake supplies essential amino acids for biomass formation, while BCAT-mediated transamination connects BCAA flux to central nitrogen and carbon metabolism - like glutamate/α-ketoglutarate balance, supporting biosynthetic pathways. Consistent with this, several malignancies preserve intracellular BCAA pools by upregulating BCAT activity or suppressing downstream BCAA catabolic flux, which can sustain mTORC1 signaling and facilitate proliferation (Ananieva and Wilkinson, 2018; Ericksen et al., 2019; Sivanand and Vander Heiden, 2020).

While BCAA metabolism is well characterized in skeletal and cardiac muscle under physiological conditions, emerging evidence suggests that tumors can reprogram BCAA utilization to support either proliferative demands or bioenergetic needs, depending on the metabolic context. Depending on tumor type, BCAA usage can be redirected in order to satisfy their energetic and anabolic needs, which in turn influences muscle metabolism (Ragni et al., 2022; Mayers et al., 2016; Sivanand and Vander Heiden, 2020). This phenomenon occurs in cancer cachexia, where muscle wasting happens simultaneously with protein and energy metabolism disruption, favoring cancer progression (Baracos et al., 2018).

Each of these three areas has been reviewed on its own. Recent reviews cover BCAA metabolism in tumors and its interaction with the immune microenvironment (Xiong et al., 2025), the catabolism of BCAA in skeletal muscle and its consequences for whole-body metabolism (Mann et al., 2021), and the role of BCAA in cardiovascular disease and heart failure (Gao and Hou, 2023; McGarrah and White, 2023). The mechanisms shared by cardiac and skeletal muscle wasting in cancer cachexia have also been described (Rausch et al., 2021). What has not been brought together is the comparison itself: how the same three amino acids are handled differently by skeletal and by cardiac muscle, how tumors reconfigure their supply, and what follows from this for each of the two tissues. The cardiac side of the question carries an additional difficulty, because most mechanistic data on myocardial BCAA catabolism come from heart failure rather than from tumor-bearing hosts, and this distinction is easily lost when the two literatures are read together. The international consensus definition of cancer cachexia notes explicitly that tissues other than skeletal muscle, cardiac muscle among them, should not be overlooked (Fearon et al., 2011).

This review therefore has three aims. The first is to contrast BCAA handling in skeletal and in cardiac muscle under physiological conditions and to identify the regulatory steps at which the two tissues differ. The second is to describe how tumors reconfigure BCAA availability and what this means for striated muscle in cancer cachexia, separating the mechanisms shared across cachexia-inducing malignancies from the features specific to individual tumor types. The third is to state, for each element of the resulting framework, whether it rests on direct measurement in a tumor-bearing host or is extrapolated from heart failure. Lung cancer and pancreatic ductal adenocarcinoma serve as the principal examples, because both are frequently complicated by cachexia (Baracos et al., 2018) and because they differ in the direction of tumor BCAA metabolism (Mayers et al., 2016); colorectal cancer is added as a shorter third example, since several of the experimental models used throughout this review derive from it. The contrast matters beyond oncology, since BCAA metabolism is a determinant of metabolic health in general (Choi et al., 2024) and prolonged BCAA supplementation supports mitochondrial biogenesis in both skeletal and cardiac muscle of healthy middle-aged male mice (D’Antona et al., 2010), whereas the same intervention in a tumor-bearing host acts on tissues whose BCAA handling has already been reconfigured.

After contrasting BCAA metabolism in skeletal and cardiac muscle (Section 2) and summarizing how tumors reprogram it (Section 3), we describe the mechanisms of striated muscle involvement shared across cachexia-inducing cancers, keeping cardiac evidence obtained in tumor-bearing hosts separate from inferences drawn from heart failure (Section 4). We then consider features specific to lung, pancreatic and colorectal cancer (Section 5), translational implications (Section 6), and the limitations of the current evidence (Section 7). What this review offers is a tissue-specific synthesis of published data together with an explicit map of what remains untested, rather than a new therapeutic or clinical model.

## 2 Differences in branched-chain amino acids metabolism

### 2.1 Skeletal muscle

As mentioned before skeletal muscle is the primary site for BCAA transamination and oxidation (**Figure 1**). The first step in catabolism of these substrates is catalyzed reversibly by BCAT. It is important to note, that the BCAT reaction operates close to equilibrium, and its net direction depends on the removal of BCKA by downstream oxidation or export from the cell. This enzyme has two isoforms: cytosolic BCAT1 (BCATc), found primarily in neurons of the central nervous system, and mitochondrial BCAT2 (BCATm), expressed in most tissues including skeletal muscle but absent from hepatocytes (Hutson et al., 2005). This process converts leucine, isoleucine and valine into their respective branched-chain alpha-keto acids (BCKA): α-ketoisocaproate (KIC), α-keto-β-methylvalerate (KMV), and α-ketoisovalerate (KIV). Apart from its main substrates, this reaction also needs a coenzyme pyridoxal 5’-phosphate (PLP), which is bound to BCAT and helps in transfer of the amino group from BCAA to α-ketoglutarate, which in turn forms glutamate and the corresponding BCKA (Hutson et al., 2005; Yennawar et al., 2001).

**Figure 1:**
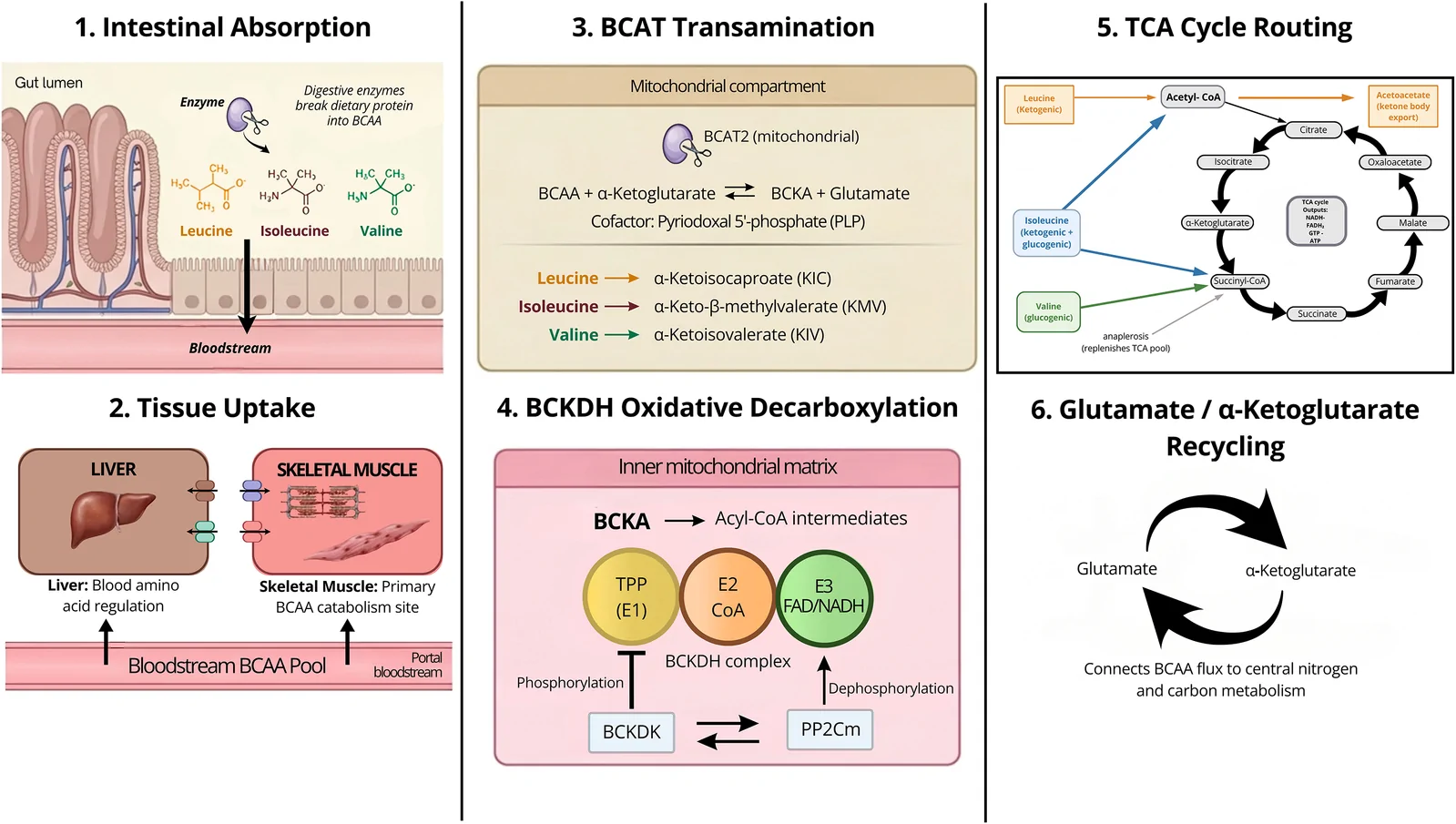
Metabolic fate of dietary branched-chain amino acids (BCAA). Schematic overview of the intestinal absorption, tissue distribution, and mitochondrial catabolism of the three branched-chain amino acids - leucine, isoleucine, and valine - and the amino acid–specific routing of their carbon skeletons into the tricarboxylic acid (TCA) cycle. 1. Intestinal absorption. Dietary protein is hydrolyzed by digestive enzymes in the gut lumen, and the liberated free BCAA are absorbed across the intestinal epithelium into the portal circulation, forming the circulating BCAA pool. 2. Tissue uptake. Circulating BCAA are taken up by peripheral tissues via membrane amino acid transporters; the liver contributes primarily to systemic blood amino acid regulation, whereas skeletal muscle is the principal site of BCAA catabolism. 3. BCAT transamination. Within the mitochondrial compartment, the mitochondrial branched-chain aminotransferase (BCAT2) catalyzes the reversible transfer of the α-amino group from each BCAA to α-ketoglutarate, using pyridoxal 5′-phosphate (PLP) as cofactor, generating glutamate and the corresponding branched-chain α-keto acids (BCKA): α-ketoisocaproate (KIC) from leucine, α-keto-β-methylvalerate (KMV) from isoleucine, and α-ketoisovalerate (KIV) from valine. Although reversible, the reaction operates close to equilibrium, and its net direction depends on the removal of BCKA by downstream oxidation or export from the cell. 4. BCKDH oxidative decarboxylation. In the inner mitochondrial matrix, the branched-chain α-keto acid dehydrogenase (BCKDH) complex (E1/E2/E3 subunits; cofactors: thiamine pyrophosphate [TPP], coenzyme A [CoA], and FAD/NAD^+^) catalyzes the irreversible, rate-limiting oxidative decarboxylation of BCKA to their respective acyl-CoA intermediates. Complex activity is reciprocally regulated by BCKDH kinase (BCKDK; phosphorylation → inactivation) and the mitochondrial phosphatase PP2Cm (dephosphorylation → reactivation). 5. TCA cycle routing. Downstream catabolism yields carbon skeletons that enter the TCA cycle in an amino acid–specific manner: leucine (ketogenic) gives rise to acetyl-CoA and the ketone body acetoacetate; isoleucine (ketogenic and glucogenic) yields both acetyl-CoA and succinyl-CoA; and valine (glucogenic) yields succinyl-CoA. Acetyl-CoA condenses with oxaloacetate to form citrate, whereas succinyl-CoA contributes anaplerotically to replenish cycle intermediates; acetoacetate is exported as a ketone body. Oxidation of cycle intermediates generates reducing equivalents (NADH, FADH_2_) and GTP/ATP. 6. Glutamate/α-ketoglutarate recycling. The glutamate produced during transamination (step 3) is reconverted to α-ketoglutarate, sustaining continuous BCAA catabolic flux and linking BCAA metabolism to central nitrogen and carbon metabolism. Color coding: leucine, orange; isoleucine, blue; valine, green. α-KG, α-ketoglutarate; ATP, adenosine triphosphate; BCAA, branched-chain amino acid; BCAT, branched-chain aminotransferase; BCAT2, mitochondrial branched-chain aminotransferase (BCATm); BCKA, branched-chain α-keto acid; BCKDH, branched-chain α-keto acid dehydrogenase; BCKDK, branched-chain α-keto acid dehydrogenase kinase; CoA, coenzyme A; E1, branched-chain α-keto acid decarboxylase; E2, dihydrolipoyl transacylase; E3, dihydrolipoamide dehydrogenase; FAD/FADH_2_, flavin adenine dinucleotide (oxidized/reduced); GTP, guanosine triphosphate; KIC, α-ketoisocaproate; KIV, α-ketoisovalerate; KMV, α-keto-β-methylvalerate; NAD^+^/NADH, nicotinamide adenine dinucleotide (oxidized/reduced); PLP, pyridoxal 5′-phosphate; PP2Cm, mitochondrial protein phosphatase 2C; TCA, tricarboxylic acid (cycle); TPP, thiamine pyrophosphate.

After that, the BCKA undergo oxidative decarboxylation catalyzed by the BCKDH complex. This multienzyme complex is regulated by two components: BCKDH kinase (BCKDK), which phosphorylates and inactivates the E1 subunit, and protein phosphatase 2C (PP2Cm), which dephosphorylates and reactivates the complex. The activity of this enzyme complex is regulated to adapt BCAA catabolism with the metabolic needs of the cell, which prevents toxic accumulation of BCKA, while still producing sufficient energy (Lu et al., 2009).

The products of this multi-stage decarboxylation differ depending on the parent BCAA. Leucine is oxidized to acetyl-CoA and acetoacetate, isoleucine to acetyl-CoA and succinyl-CoA, and valine generates succinyl-CoA only. This is the reason why leucine is called a ketogenic amino acid, valine a glucogenic one and isoleucine falls into both categories.

These products feed directly into the tricarboxylic acid (TCA) cycle, where they serve as intermediates to generate reducing equivalents. Succinyl-CoA, formed from isoleucine and valine, contributes to anaplerotic reactions, replenishing TCA cycle intermediates that may be depleted during prolonged metabolic activity (Mann et al., 2021).

Physiologically, this catabolic pathway operates alongside a large anabolic demand, because skeletal muscle is the largest body protein reservoir and a major determinant of whole-body BCAA fate. A substantial portion of dietary BCAA are retained for myofibrillar protein turnover and accumulation in the fed state, whereas during fasting, prolonged exercise, or systemic stress - skeletal muscle increases BCAA oxidative flux to support mitochondrial energy production and nitrogen turnover - which shifts BCAA away from incorporation into protein (Shimomura et al., 2004; Wolfe, 2017). Thus, skeletal muscle BCAA metabolism is best interpreted as state-dependent partitioning between anabolic retention and oxidative disposal, coordinated by regulation of the BCAT/BCKDH axis to match substrate use to physiological demand while limiting accumulation of BCAA-derived keto acids (Hutson et al., 2005; Lu et al., 2009).

### 2.2 Cardiac muscle

Cardiomyocytes predominantly utilize glucose and fatty acids as their primary metabolic substrates to meet the energetic demands of continuous contractile activity (Ng et al., 2023). The healthy myocardium shows relatively lower rates of BCAA oxidation in the TCA cycle when compared to other key sites of BCAA breakdown, particularly skeletal muscle. In addition, concerning total mass, cardiac tissue has a minor influence on BCAA oxidation, as the main sites for these metabolic processes are skeletal muscle and fat tissue (McGarrah and White, 2023). Under physiological circumstances, branched-chain amino acids account for less than 2% of total ATP synthesis in a healthy heart (Ng et al., 2023).

BCAA are transported into cardiomyocytes via specific amino acid transporters, whose activity is modulated by substrate availability (Ng et al., 2023). As in skeletal muscle, they are catabolized by BCAT2 and the BCKDH complex, whose activity is set by the balance between BCKDK and PP2Cm (encoded by *PPM1K*). PP2Cm exhibits elevated expression levels within heart, brain and liver, aligning with the detected activity of branched chain ketoacid dehydrogenase complex in these specific tissues (Lu et al., 2009).

As previously discussed, BCAA catabolism produces acetyl-CoA and succinyl-CoA, which fuel the tricarboxylic acid cycle. In cardiac tissue, this contribution is modest under physiological conditions, but its impairment can disturb mitochondrial redox balance in the failing heart (Ananieva and Wilkinson, 2018; Huang et al., 2011).

The main anabolic relevance of BCAA in cardiomyocytes is best described as continuous protein homeostasis - ongoing renewal of contractile and mitochondrial proteins required to sustain function under constant heart workload - together with strict control of BCAA catabolic flux to prevent accumulation of BCAA-derived intermediates that can negatively affect mitochondrial performance and redox balance (Huang et al., 2011; Sun et al., 2016).

## 3 Tumor-intrinsic reprogramming of BCAA metabolism

Cancer cells exhibit enhanced uptake of branched-chain amino acids which serve dual roles in supporting tumor growth. In the process of protein biosynthesis, these amino acids are crucial, and they additionally serve as substrates that facilitate energy production through oxidative metabolic mechanisms. Recognizing that branched-chain amino acids (BCAA) are not produced within the body, tumor tissues depend on external dietary input and proteolytic breakdown to meet their metabolic necessities (Ananieva and Wilkinson, 2018). Through proteolytic breakdown they become available for catabolism in BCAT-expressing tissues, particularly skeletal muscle. This metabolic process generates branched-chain keto acids that enter circulation (Sun et al., 2016). Recent studies have revealed that the initial enzymatic step in BCAA catabolism, mediated by the cytosolic (BCAT1) and mitochondrial (BCAT2) branched-chain aminotransferases, is frequently upregulated in malignancies (Xu et al., 2023). These enzymes facilitate the transamination process of branched-chain amino acids (BCAA) into their respective α-keto acids, concurrently transforming α-ketoglutarate into glutamate, thus establishing a connection between BCAA metabolism and the central carbon and nitrogen metabolic pathways (Ananieva and Wilkinson, 2018).

In several tumor types, BCAT1 is upregulated and BCAA catabolism supports tumor growth. BCAT1 overexpression has been linked to proliferation in head and neck squamous cell carcinoma (Wang et al., 2021). In melanoma cells BCAT1 knockdown suppressed proliferation and migration and reduced oxidative phosphorylation (Zhang et al., 2021). In breast cancer, BCAT1 and other BCAA catabolic enzymes were upregulated in tumor tissue, and in breast cancer cell lines BCAT1 promoted growth and colony formation through mTOR-dependent mitochondrial biogenesis (Zhang and Han, 2017). In gliomas, BCAT1 expression was restricted to tumors with wild-type IDH1 and IDH2, and its suppression reduced glutamate excretion, proliferation and invasiveness *in vitro*, as well as tumor growth in a glioblastoma xenograft model (Tönjes et al., 2013). These examples of enhanced BCAA catabolism contrast with the suppression of BCAA catabolism observed across many other malignancies, and illustrate that the direction of BCAA metabolic reprogramming is tumor-type dependent.

Pan-cancer analyses indicate that approximately 70% of malignancies display significant suppression of BCAA metabolism (Ericksen et al., 2019). The inhibition of branched-chain amino acid (BCAA) catabolism within neoplasms culminates in the intracellular enrichment of these amino acids, thereby perpetuating the activation of mechanistic target of rapamycin complex 1 (mTORC1), which serves as a crucial modulator of tumor cell proliferation. The intricate triggering of mTORC1 relies heavily on having both mitogenic signals and sufficient nutritional inputs, especially branched-chain amino acids (Sivanand and Vander Heiden, 2020). Malignant cells frequently achieve persistent mTORC1 signaling through two complementary mechanisms: genetic alterations in regulatory components (such as TSC1/TSC2 deletions) and metabolic reprogramming that preserves BCAA pools by reducing their catabolic flux. Together, these two mechanisms sustain proliferative signaling and preserve metabolic flexibility during nutrient deprivation (Ericksen et al., 2019; Sivanand and Vander Heiden, 2020).

Furthermore, BCAA metabolic reprogramming varies significantly across cancer types, with divergent expression and activity of key enzymes leading to different pathway activation or suppression. Tissue origin affects how cancers metabolize branched-chain amino acids in unique ways. Non-small cell lung cancer (NSCLC) actively incorporates circulating BCAA into cellular proteins and exploits them as a nitrogen source for nucleotide synthesis, driving increased BCAA uptake. By contrast, pancreatic ductal adenocarcinoma (PDAC) tumors take up less BCAA and rely more on alternative nitrogen sources (Mayers et al., 2016). However, increased circulating levels of branched-chain amino acids are observed during subclinical stage (2–5 years before diagnosis) in patients with pancreatic cancer. This increment is a result of an overflow of proteolytic operations in peripheral tissues, exceeding the total usage of branched-chain amino acids (BCAA). Boosted levels of BCAA could function as an informative predictive biomarker in models of pancreatic cancer risk stratification when looked at with additional clinical assessments (Mayers et al., 2014; Sivanand and Vander Heiden, 2020).

## 4 Shared mechanisms of skeletal and cardiac muscle involvement in cancer cachexia

A useful framework is that cardiac and skeletal muscle wasting share common “modules”: inflammatory cytokine exposure, activation of ubiquitin–proteasome/autophagy machinery, mitochondrial dysfunction with ROS amplification, and endocrine/nutrient shifts producing metabolic inflexibility. These modules can be synchronized by circulating factors and tissue-derived signals rather than acting independently in each organ (Rausch et al., 2021). Because these modules are common to the malignancies considered in this review, they are described once below, first for skeletal and then for cardiac muscle (**Figure 2**), whereas features specific to individual tumor types are considered in Section 5.

**Figure 2:**
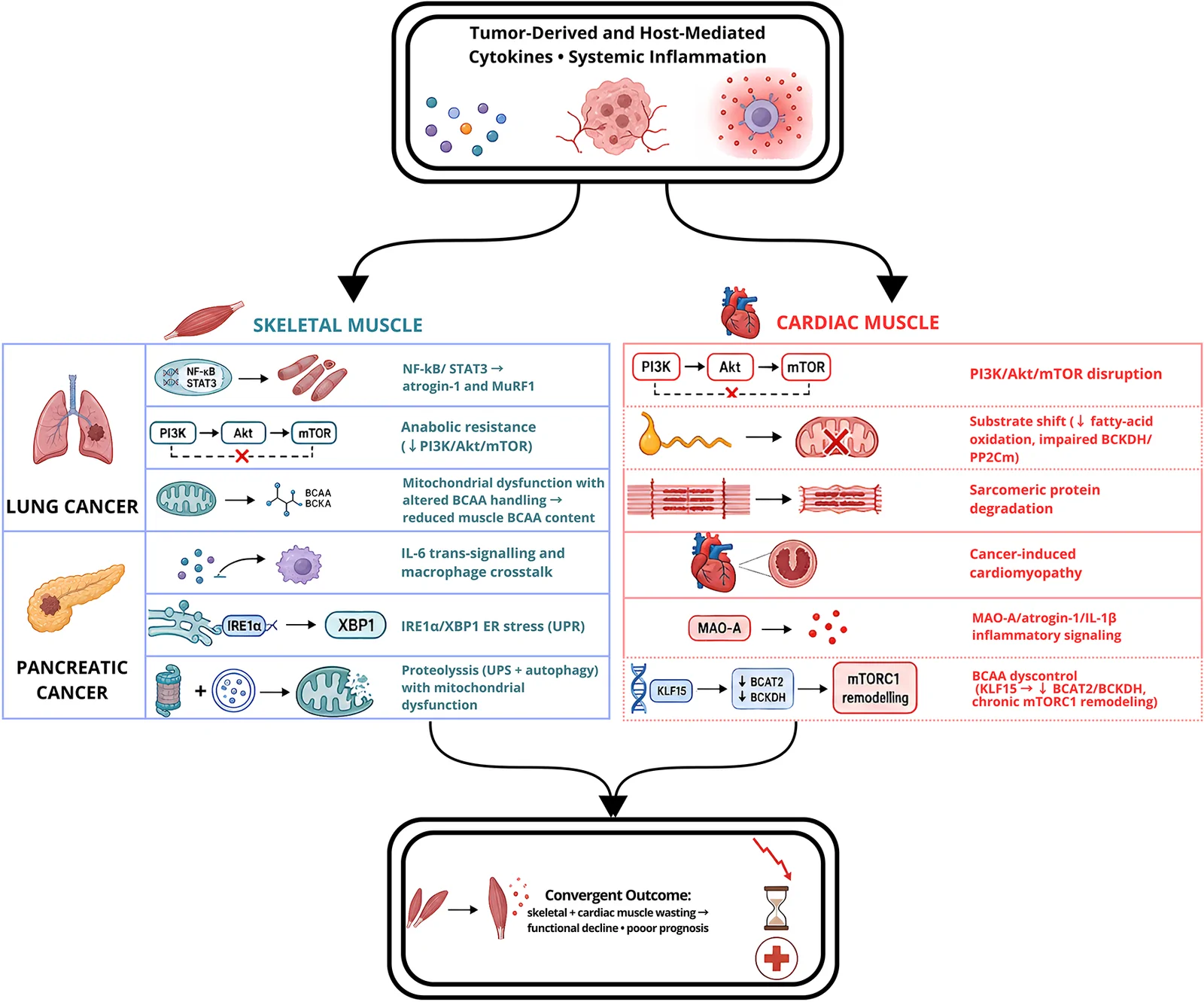
Convergent and cancer-specific pathways of BCAA dysmetabolism in skeletal and cardiac muscle wasting. Comparative matrix contrasting lung cancer (top row) and pancreatic ductal adenocarcinoma (PDAC; bottom row) across skeletal muscle (left, teal) and cardiac muscle (right, red). A shared upstream of tumor-derived and host-mediated cytokines with systemic inflammation initiates catabolic signaling across both tissues and both malignancies. In lung cancer, skeletal muscle is driven by NF-κB/STAT3-mediated atrogene induction, anabolic resistance (impaired PI3K/Akt/mTOR), and mitochondrial dysfunction with altered BCAA handling and reduced muscle BCAA content, whereas the myocardium shows reduced mTOR signaling, a substrate shift away from fatty-acid oxidation, sarcomeric protein degradation and, by extrapolation from heart failure models, impaired BCKDH/PP2Cm control. In PDAC, skeletal muscle is shaped by IL-6 trans-signaling and macrophage crosstalk, IRE1α/XBP1 ER stress, and proteolysis with mitochondrial dysfunction, while the heart shows cancer-induced cardiomyopathy, MAO-A-associated inflammatory and atrophy signaling, and BCAA dyscontrol (KLF15-dependent suppression of BCAT2/BCKDH, extrapolated from heart failure models). Despite distinct upstream drivers, both cancers converge on combined skeletal and cardiac muscle wasting, functional decline, and poor prognosis. Elements extrapolated from heart failure models are outlined with dashed borders. The cardiac findings shown in the lung cancer row were obtained in colorectal tumor models (*Apc^Min/+^* and C26) and, for BCAA catabolic control, in heart failure models; no direct cardiac data from lung cancer models were identified (Section 5.1). Atrogin-1, muscle atrophy F-box protein; BCAA, branched-chain amino acid; BCAT2, mitochondrial branched-chain aminotransferase; BCKA, branched-chain α-keto acid; BCKDH, branched-chain α-keto acid dehydrogenase; IL-1β, interleukin-1 beta; IL-6, interleukin-6; IRE1α, inositol-requiring enzyme 1 alpha; KLF15, Krüppel-like factor 15; MAO-A, monoamine oxidase A; mTOR(C1), mechanistic target of rapamycin (complex 1); MuRF1, muscle RING-finger protein 1; NF-κB, nuclear factor kappa B; PP2Cm, mitochondrial protein phosphatase 2C; STAT3, signal transducer and activator of transcription 3; UPS, ubiquitin–proteasome system; XBP1, X-box binding protein 1.

### 4.1 Skeletal muscle

The cachectic process is driven by a systemic pro-inflammatory state, initiated and maintained by tumor-derived and host-derived cytokines. The concentrations of interleukin-6 (IL-6), tumor necrosis factor-α (TNF-α), and interferon-γ (IFN-γ) are among the principal mediators identified in cell culture and tumor xenograft models, although confirmatory data from patients remain limited (Baracos et al., 2018). These cytokines activate transcription factors such as NF-κB and STAT3 in myocytes, leading to the transcriptional upregulation of muscle-specific ubiquitin ligases, including atrogin-1 and MuRF1, both of which mediate protein degradation through the ubiquitin-proteasome system. At the same time, there exists an involvement of autophagy-lysosomal pathways, particularly in type II muscle fibers, which accelerates the catabolism of cellular components as a reaction to stressors (Webster et al., 2020).

Importantly, this enhanced proteolysis is not balanced by corresponding increases in protein synthesis. The signaling network featuring PI3K/Akt/mTOR, integral for muscle hypertrophy, is commonly affected in the scenario of cachexia. In alignment with the phenomenon of anabolic resistance observed in cancer cachexia, the mechanistic target of rapamycin complex 1 (mTORC1) and the subsequent pathways involved in protein synthesis exhibit a diminished responsiveness, even in the presence of upstream anabolic stimuli and nutritional signals, thereby suggesting an impairment in the transduction of anabolic signals (Hardee et al., 2017). Recognizing the fundamental importance of mTORC1 in regulating ribosomal biogenesis and protein translation, its suppression causes a drop in contractile protein synthesis, leading to increased muscle atrophy (Geremia et al., 2022).

Overlaying these impairments is a disruption of amino acid metabolism, with BCAA playing a central role in maintaining muscle energy balance and anabolic signaling. In tumor-bearing mice, skeletal muscle BCAA content is reduced: in the C26 model, BCAA concentrations were reduced in several skeletal muscles together with lower expression of the BCAA transporter LAT1 (Zerrouk et al., 2026). In mice bearing Lewis lung carcinoma, muscle-specific deletion of *Bckdk*, which is expected to relieve the inhibition of BCKDH, lowered muscle and serum BCAA concentrations, reduced mTOR signaling, and accelerated the onset of cachexia and death, whereas a BCAA-enriched diet prolonged survival in the knockout mice but not in tumor-bearing wild-type mice (Chen et al., 2024). In cachectic muscle tissue, this metabolic imbalance is further intensified by cytokines that promote inflammation, which attenuate mTORC1 activation even when leucine availability is adequate, although this resistance could be partly overcome in patients by a high-protein, leucine-enriched supplement. This reflects a breakdown in nutrient-sensing and anabolic pathways that are essential for preserving muscle mass and mitochondrial integrity (Ananieva and Wilkinson, 2018; Hardee et al., 2017).

Simultaneously, cancer-associated inflammation induces mitochondrial dysfunction, which contributes to a shift in muscle energy metabolism. During cachexia, mitochondria in muscle cells show reduced oxidative phosphorylation, lower adenosine triphosphate (ATP) synthesis and increased production of reactive oxygen species (ROS) (Ragni et al., 2022). Particularly, mitophagy - the deliberate breakdown of nonfunctioning mitochondria is increased and occurs alongside reduced mitochondrial biogenesis, together diminishing mitochondrial content and aggravating oxidative stress, as shown in a murine pancreatic cancer cachexia model (Gicquel et al., 2024). This perturbation is clearly observable in skeletal muscle biopsies obtained from cachectic individuals, wherein deviations in mitochondrial architecture, membrane potential, and enzymatic functionality have been recorded (Webster et al., 2020).

### 4.2 Cardiac muscle: direct evidence and extrapolation from heart failure

Although cancer cachexia is primarily associated with skeletal muscle wasting, a growing body of evidence indicates that the heart is not spared from its systemic consequences. The secondary consequences of cancer on cardiac myocytes stem from a convergence of persistent inflammation, modified nutrient accessibility, and augmented physiological demands, all of which collectively undermine myocardial performance and energy metabolism (Baracos et al., 2018). Though the myocardium may not have the same scale of mass loss as skeletal muscle, cachexia results in metabolic transformations that amplify its risk for functional impairments.

As mentioned before in relation to skeletal muscle, pro-inflammatory cytokines, namely TNF-α, IL-6, and IL-1β, similarly cause harmful effects to cardiac tissue. In the setting of cancer cachexia, their chronic elevation contributes to a sustained catabolic environment. In the myocardium of cachectic *Apc^Min/+^* mice, a model of intestinal tumorigenesis, reduced mTOR signaling with diminished phosphorylation of its downstream targets has been reported - independently of Akt activation. mTOR signaling is essential for maintaining protein turnover, mitochondrial integrity, and adaptive hypertrophy. Its suppression in cachectic conditions may impair the heart’s ability to maintain contractile function and respond to physiological stressors, ultimately increasing the risk of myocardial dysfunction (Manne et al., 2013; Webster et al., 2020).

Furthermore, catabolic signaling cascades recognized for their activity in skeletal muscle during cachexia - such as the ubiquitin-proteasome pathway and autophagy - are similarly upregulated within the myocardium (Rausch et al., 2021), although this pattern is model-dependent: in *Apc^Min/+^* hearts protein ubiquitination was not increased, and autophagy markers together with reduced myofibrillar protein synthesis predominated (Manne et al., 2013). In C26 tumor-bearing mice, increased cardiac expression of MuRF1 (mRNA and protein) and atrogin-1 (mRNA) was accompanied by increased protein ubiquitination and by reductions in myosin heavy chain and troponin I of 43% and 58%, respectively (Tian et al., 2011). Persistent activation of these pathways leads to degradation of sarcomeric proteins and of structural components required for effective electromechanical coupling (Rausch et al., 2021). In the context of cancer cachexia, the cardiac muscle experiences both structural and metabolic alterations, encompassing a depletion of myofibrillar and sarcomeric components, in conjunction with mitochondrial dysfunction; these modifications are concomitant, in preclinical models, with diminished ejection fraction and reduced exercise capacity (Rausch et al., 2021; Tichy and Parry, 2023).

Substrate utilization in the cachectic myocardium is less well characterized. Under physiological conditions, the adult heart relies predominantly on fatty acid oxidation to meet its high energy demand. In heart failure, reduced fatty acid oxidation is accompanied by a shift toward glucose utilization, reflecting reactivation of a fetal-like metabolic program (Sun et al., 2016). Increased ketone body utilization is generally regarded as a separate adaptation of the failing myocardium (Ng et al., 2023). A partly similar pattern has been described in cancer cachexia: in hearts of cachectic C26 tumor-bearing mice, expression of PPARα and its target gene *CPT1β* was reduced, and myosin heavy chain and glucose transporter expression shifted from adult toward fetal isoforms (Tian et al., 2011). These are expression-level changes and the authors noted that a reduced capacity for fatty acid oxidation still requires confirmation by activity measurements. Thus, its consequences for myocardial ATP supply in cachexia remain to be established.

Mechanistic evidence linking BCAA catabolism to cardiac dysfunction originates primarily from heart failure models. In pressure-overloaded mouse hearts and in human dilated cardiomyopathy, genes of the BCAA catabolic pathway, including BCAT2, BCKDH subunits and the BCKDH phosphatase PP2Cm, are downregulated. Loss of PP2Cm without a corresponding decrease in BCKDK increases inhibitory phosphorylation of BCKDH (Huang et al., 2011; Lu et al., 2009; Sun et al., 2016). In these hearts, the resulting defect led to intramyocardial accumulation of BCKA, whereas tissue BCAA levels were largely unchanged, and in isolated cardiac mitochondria BCKA inhibited complex I-dependent respiration and increased superoxide production (Sun et al., 2016). Other heart failure studies, however, report accumulation of both BCAA and BCKA, linked to persistent mTOR signaling, cardiac hypertrophy and insulin resistance (McGarrah and White, 2023; Ng et al., 2023). The involvement of BCAA catabolic defects in the cachectic myocardium should therefore be regarded as a plausible hypothesis extrapolated from heart failure rather than as an established mechanism of cancer cachexia.

In pressure-overloaded mouse hearts, KLF15 expression is reduced, and KLF15 was identified as a key upstream regulator of BCAT2, BCKDH subunits and PP2Cm. Its loss is therefore a potential mechanism of the coordinated suppression of BCAA catabolism in the failing heart (Sun et al., 2016). A recent review of cardiovascular BCAA dysregulation indicates that an imbalance in BCAA catabolism may contribute to cardiac dysfunction (Yin et al., 2026), yet it addresses cardiovascular disease rather than cancer cachexia. It is still unclear whether the KLF15–BCAA catabolic axis is suppressed in the heart of tumor-bearing hosts.

Two kinds of evidence on cardiac involvement need to be distinguished. Direct evidence comes from tumor-bearing hosts. Cardiac atrophy with proteolytic activation has been documented in C26 tumor-bearing mice (Tian et al., 2011) and in KPC mice with pancreatic carcinoma (Stelter et al., 2024), while reduced mTOR signaling, independent of Akt, was reported in *Apc^Min/+^* mice (Manne et al., 2013). Systolic and diastolic dysfunction accompanied cardiac atrophy in murine and patient-derived pancreatic cancer models and in an ovarian cancer model (Ogilvie et al., 2024), whereas in patients with advanced cancer low left ventricular mass occurred with preserved ejection fraction (Anker et al., 2025). In contrast, the mechanistic data linking BCAA catabolism to cardiac dysfunction come from genetic, pressure-overload and heart failure models (Huang et al., 2011; Lu et al., 2009; Sun et al., 2016). To our knowledge, myocardial BCKDH activity, PP2Cm expression and intramyocardial BCKA content have not yet been reported in a cancer cachexia model (see Section 7).

Taken together, these findings indicate that the heart is affected in cancer cachexia, although indirectly. While the myocardium does not undergo the dramatic mass loss seen in skeletal muscle, its function may nonetheless be compromised by a combination of metabolic inflexibility, excessive mechanical demand and, by extrapolation from heart failure, impaired BCAA catabolism. This may eventually lead to subclinical cardiac dysfunction that can potentially develop into evident heart failure if not spotted or addressed suitably.

## 5 Features specific to lung, pancreatic and colorectal cancer

### 5.1 Lung cancer

Lung cancer has systemic effects, and cancer cachexia is among the most clinically important of them: a syndrome characterized by the profound and involuntary loss of skeletal muscle mass. Cachexia affects approximately 50% of patients with lung cancer, defined by a body-weight loss exceeding 5% within the prior six months, and it demonstrates resistance to nutritional interventions, in contrast to starvation, due to its complex etiological factors (Baracos et al., 2018). This progressive wasting condition emerges from the complex interactions of metabolic and inflammatory irregularities that collectively disturb the balance of skeletal muscle stability (Webster et al., 2020).

A unique aspect of lung cancer induced skeletal muscle dysfunction is the role played by respiratory compromise. As the primary organ compromised by pathology, the functional deterioration of the pulmonary system leads to enduring hypoxemia and dyspnea, conditions that significantly impair the individual’s ability to engage in physical exertion.

Muscle disuse serves as a distinct factor in the etiology of atrophy: extended periods of physical inactivity initiate muscle protein catabolism pathways and enhance muscle insulin resistance, thereby expediting the deterioration of muscle mass and functionality (Lavalle et al., 2024). Lung cancer patients often exhibit reduced muscular strength alongside sarcopenia, which have separate and important prognostic meanings (Cereda et al., 2022; Wang et al., 2023).

These events result in a vicious cycle: systemic inflammation promotes catabolism and suppresses anabolism; respiratory insufficiency reduces muscle loading and contractile activity; and metabolic inefficiencies disrupt energy and amino acid homeostasis. The interplay of these dependent processes encourages the reduction of skeletal muscle mass, culminating in lower mobility, heightened treatment challenges, and a generally negative prognosis (**Table 1**). Despite the devastating clinical impact of muscle loss in lung cancer, current therapeutic strategies remain largely supportive, emphasizing the urgent need for interventions that target these intersecting molecular pathways to preserve muscle function and improve quality of life.

**Table 1:** Primary experimental and clinical evidence on BCAA metabolism and muscle involvement specific to lung cancer.

Finding	Model or population	Ref.
Preclinical evidence		
Non-small cell lung cancer tumors take up circulating BCAA and use BCAA-derived nitrogen for biosynthesis; BCAA uptake is higher than in PDAC tumors	Autochthonous mouse models of *Kras*-driven NSCLC and PDAC	(Mayers et al., 2016)
Muscle-specific deletion of *Bckdk* lowered muscle and serum BCAA, reduced mTOR signaling, accelerated the onset of cachexia and shortened survival	Mice bearing Lewis lung carcinoma	(Chen et al., 2024)
A BCAA-enriched diet delayed cachexia and prolonged survival in *Bckdk*-cKO mice, but did not change survival in tumor-bearing wild-type mice	Muscle-specific *Bckdk*-cKO and wild-type mice bearing Lewis lung carcinoma	(Chen et al., 2024)
Clinical evidence		
Sarcopenia is frequent in lung cancer inpatients and is associated with frailty, nutritional risk and malnutrition	Hospitalized patients with lung cancer	(Wang et al., 2023)
Muscle weakness adds prognostic information to sarcopenia grading	Patients with cancer	(Cereda et al., 2022)

Only primary research is listed. Mechanisms shared across cachexia-inducing cancers are described in Section 4 and are not repeated here. Direct evidence on the cachectic heart was obtained in colorectal, pancreatic and ovarian tumor models and in mixed cohorts of patients with advanced cancer (Section 4.2, **Table 2**), not in lung cancer models.

The mechanical consequences of systemic muscle loss further compound this metabolic burden. As lung cancer progresses and skeletal muscle atrophy impairs mobility and physical conditioning, the heart must sustain adequate perfusion with reduced assistance from peripheral musculature. This culminates in an augmented heart rate, an elevated preload, and an increased demand for contractility - all of which transpire within a context of diminished metabolic efficiency and limited substrate availability (Tichy and Parry, 2023). The mismatch between energy demand and mitochondrial capacity produces a persistent energy deficit that impairs calcium cycling and contractile force, reducing cardiac efficiency.

At the level of BCAA metabolism, lung cancer combines avid uptake of circulating BCAA by non-small cell lung cancer, described in Section 3 (Mayers et al., 2016), with genetic evidence, described in Section 4.1, that muscle BCAA catabolism modifies the course of cachexia in mice bearing Lewis lung carcinoma (Chen et al., 2024). By contrast, the direct evidence on the cachectic heart summarized in Section 4.2 was obtained in colorectal, pancreatic and ovarian tumor models and in mixed cohorts of patients with advanced cancer, not specifically in lung cancer; cardiac involvement in lung cancer cachexia is therefore inferred from these data.

### 5.2 Pancreatic ductal adenocarcinoma

Pancreatic ductal adenocarcinoma (PDAC) is among the most cachexia-prone malignancies, and its systemic metabolic phenotype strongly affects striated muscle tissues. PDAC intensifies tumor demand for essential amino acids and reprogram host nutrient partitioning, creating a state of persistent amino-acid redistribution rather than a simple dietary deficiency. Inflammatory and endocrine disturbances accompany this competition for nutrients, suppressing the anabolic response and sustaining catabolic signaling in several tissues at once.

Skeletal muscle wasting in PDAC often begins early and is frequently accompanied by myosteatosis, with these body-composition changes associating with worse treatment tolerance and survival. Importantly, PDAC cachexia involves coordinated changes across several tissues rather than passive loss of muscle mass: muscle adapts its gene expression, substrate selection, and stress signaling under sustained tumor-derived pressure, and these adaptations become maladaptive when chronically engaged (Compton et al., 2024).

PDAC increases tumor uptake and use of BCAA and reshapes mitochondrial BCAA catabolism. Experimental work supports that PDAC growth can be sustained by BCAA-dependent metabolic programs, including lipid-metabolic outputs, with regulation of key BCAA-catabolic machinery (e.g., BCAT2/BCKDH-related control) impacting tumor biology (Lee et al., 2019; Lei et al., 2020). This diversion of BCAA toward the tumor matters for muscle, because skeletal muscle is normally a dominant site of BCAA transamination and systemic BCAA nitrogen trafficking; when tumor demand rises and systemic inflammation is high, muscle BCAA handling and anabolic signaling become unreliable even if intake is adequate (Mann et al., 2021).

A PDAC-specific driver that links tumor, adipose tissue, and muscle is the IL-6 trans-signaling loop. In patient-derived orthotopic models recapitulating PDAC cachexia heterogeneity, tumor-derived IL-6 promotes adipose inflammation and lipolysis, releasing fatty acids that accumulate in skeletal muscle (myosteatosis) and amplify metabolic stress. Skeletal muscle concurrently increases IL-6 receptor components and contributes soluble IL-6 receptor (sIL-6R), enabling IL-6 trans-signaling that reinforces catabolic programs. Disruption of tumor IL-6 attenuates muscle lipid accumulation, metabolic disturbance, and atrophy, supporting a causal rather than purely correlative role for the IL-6/sIL-6R axis in PDAC muscle loss (Rupert et al., 2021).

Inflammation in PDAC cachexia is further shaped by immune–tumor crosstalk, which can amplify systemic wasting signals. Macrophage-cancer cell interactions potentiate cachexia-associated signaling, linking the tumor microenvironment to whole-body catabolism (Liu et al., 2024). Downstream, skeletal muscle activates the ubiquitin-proteasome and autophagy-lysosomal pathways, with loss of contractile proteins and progressive weakness.

At the intracellular level, chronic stress-adaptation pathways are increasingly recognized as central nodes in PDAC muscle biology. The IRE1α-XBP1 arm of the unfolded protein response is engaged in PDAC cachexia and promotes expression of proteolysis-related genes (including autophagy and ubiquitin–proteasome components), enhances inflammatory JAK–STAT3 signaling, and reshapes metabolic gene programs. Muscle-specific deletion of *XBP1* attenuates tumor-induced atrophy, and pharmacologic inhibition of IRE1α reduces cachexia molecular signatures while improving muscle mass and strength - which suggests that nutritional support alone is unlikely to be sufficient unless inflammatory and stress signaling is addressed in parallel (Joshi et al., 2025).

Mitochondrial dysfunction is a core feature of the PDAC skeletal-muscle phenotype. Multi-omics analyses in murine PDAC-induced cachexia models demonstrate reduced oxidative ATP production, reduced activity and abundance of respiratory complexes, altered mitochondrial morphology, and greater reliance on glycolysis; amino-acid metabolism pathways are among those prominently dysregulated (Gicquel et al., 2024). Impaired mitochondrial function limits the oxidation of amino-acid-derived substrates while inflammation continues to drive proteolysis, intensifying energetic inefficiency and weakening the normal anabolic use of BCAA.

In parallel, pro-inflammatory cytokines and angiotensin II promote muscle protein breakdown through the ubiquitin-proteasome system, while inflammatory signaling concurrently suppresses anabolic PI3K/Akt/mTOR activity, resulting in a net negative protein balance (Miyamoto et al., 2016; Sanders et al., 2005). In this context, perturbations in BCAA availability and handling amplify vulnerability to atrophy by reducing anabolic input and by interacting with mitochondrial stress. Additionally, BCAA availability in PDAC also affects lipid balance: in PDAC cells *in vitro*, BCAA deprivation can suppress mitochondrial fatty-acid import (e.g., via CPT1-related control) and redirect lipid flux toward triglyceride synthesis and lipid droplet accumulation (Gotvaldová et al., 2024). Reports on BCAT2 in PDAC are not consistent: functional studies describe increased expression supporting tumor growth (Lee et al., 2019; Lei et al., 2020), whereas pan-cancer transcriptomic profiling reports reduced expression in PAAD (Cao et al., 2024) and autochthonous tumors show reduced BCAA uptake (Mayers et al., 2016).

Although skeletal muscle loss is the most visible hallmark of PDAC cachexia, the myocardium is increasingly recognized as a target of the same systemic program. PDAC creates a milieu of persistent inflammation and altered nutrient availability (often complicated by malabsorption and endocrine dysfunction), in which the heart must sustain continuous work while metabolic flexibility is eroded (Compton et al., 2024; Tichy and Parry, 2023). Subclinical cardiac impairment worsens fatigue and exercise intolerance and increases vulnerability during treatment.

In two pancreatic cancer models (a murine PDAC model and a patient-derived xenograft) and in an ovarian cancer model, experimental evidence supports a cancer-induced cardiomyopathy phenotype characterized by cardiac atrophy, functional impairment, and metabolic derangements consistent with impaired mitochondrial energetics and substrate utilization (Ogilvie et al., 2024). Myocardial BCAA catabolism contributes anaplerotic input to the TCA cycle and supports redox balance, so remodeling that impairs oxidative metabolism may also affect BCAA flux, even though BCAA are not a major cardiac fuel at baseline.

PDAC-specific model data strengthen the link between pancreatic cancer and cardiac wasting. In transgenic mice with pancreatic carcinoma, cardiac cachexia has been associated with increased MAO-A expression, upregulation of atrogin-1, and elevated IL-1β in myocardial tissue. MAO-A inhibition with harmine largely reversed these inflammatory and atrophy-related changes, although cardiac function and cardiomyocyte size were not assessed and the study included no sham control (Stelter et al., 2024). These findings align with broader cachexia biology, where inflammatory and proteolytic activation collaborate to impair excitation–contraction coupling and degrade contractile structures.

Clinical data are now available. Clinical and echocardiographic profiling in advanced cancer populations demonstrates structural and functional signatures consistent with cardiac tissue loss (“cardiac wasting”) detectable with standard imaging, with reduced chamber volumes and stroke volume but preserved left ventricular ejection fraction. While not PDAC-exclusive, these observations are directly relevant because PDAC is among the malignancies most frequently complicated by cachexia (Baracos et al., 2018) and because they support the clinical reality that myocardial involvement can be measurable and clinically meaningful (Anker et al., 2025).

Taken together, PDAC-associated cachexia should be considered a multi-organ striated-muscle syndrome (**Table 2**).

**Table 2:** Primary experimental and clinical evidence on BCAA metabolism and striated muscle involvement specific to pancreatic ductal adenocarcinoma.

Finding	Model or population	Ref.
Preclinical evidence - tumor		
PDAC tumors take up less BCAA than non-small cell lung cancer tumors and rely on alternative nitrogen sources	Autochthonous mouse models of *Kras*-driven PDAC and NSCLC	(Mayers et al., 2016)
PDAC cells take up more BCAA than non-transformed pancreatic ductal cells; knockdown of BCAT2 or of BCKDHA impairs proliferation and fatty-acid synthesis, and BCKDHA knockdown reduces tumor growth	Human PDAC cell lines and subcutaneous xenografts in mice	(Lee et al., 2019)
Acetylation of BCAT2 at K44 promotes its degradation and suppresses BCAA catabolism; the non-acetylatable mutant accelerates proliferation and tumor growth on both normal and BCAA-restricted diets, with a more pronounced effect under restriction	Human PDAC cell lines and subcutaneous xenografts in mice fed a normal or a BCAA-restricted diet	(Lei et al., 2020)
BCAA deprivation suppresses mitochondrial fatty-acid import and redirects lipid flux toward triglyceride synthesis and lipid droplets	Three human PDAC cell lines, *in vitro* only	(Gotvaldová et al., 2024)
Preclinical evidence – host tissues		
Tumor-derived IL-6 and IL-6 trans-signaling drive adipose and skeletal muscle wasting; disrupting tumor IL-6 attenuates muscle lipid accumulation and atrophy	Orthotopic and patient-derived mouse models of PDAC	(Rupert et al., 2021)
Tumor-derived CCL2 recruits and reprograms macrophages, which in turn drive tumor TWEAK secretion and MuRF1-mediated muscle atrophy; macrophage depletion reverses muscle wasting	Orthotopic KPC allografts in wild-type and *Ccr2*-deficient mice, tumor–macrophage co-cultures and patient tumor samples	(Liu et al., 2024)
IRE1α/XBP1 activation promotes proteolysis, autophagy and inflammation in skeletal muscle; muscle-specific *XBP1* deletion or IRE1α inhibition preserves muscle mass and strength	Mouse models of PDAC cachexia	(Joshi et al., 2025)
Reduced oxidative ATP production, impaired respiratory complex activity and altered mitochondrial morphology in skeletal muscle	Murine PDAC-induced cachexia model, multi-omics	(Gicquel et al., 2024)
Cardiac atrophy, contractile dysfunction and impaired mitochondrial energetics	Murine PDAC model, PDAC patient-derived xenograft and an ovarian cancer model	(Ogilvie et al., 2024)
Increased myocardial MAO-A protein and increased fractions of atrogin-1+, IL-1β+ and TNF+ cells; MAO-A inhibition with harmine reversed these changes	Transgenic (KPC) mice with pancreatic carcinoma	(Stelter et al., 2024)
BCAA content was reduced in lungs and spleen, and valine in the pancreas, but not in the heart; myocardial glutamate was reduced	Human PDAC xenograft in mice	(Sharma et al., 2025)
Clinical evidence		
Circulating BCAA are elevated 2 to 5 years before diagnosis	Prospective human cohorts	(Mayers et al., 2014)
Echocardiographic and clinical signatures of cardiac wasting are detectable with standard imaging, with reduced chamber volumes and stroke volume but preserved LVEF	Patients with advanced cancer	(Anker et al., 2025)

Only primary research is listed. Mechanisms shared across cachexia-inducing cancers are described in Section 4 and are not repeated here. Findings extrapolated from heart failure and from genetic models of impaired BCAA catabolism (Huang et al., 2011; Lu et al., 2009; Sun et al., 2016; Yin et al., 2026) are identified as such in Section 4.2 and are not listed here.

### 5.3 Colorectal cancer

Colorectal cancer is included because several experimental findings summarized in Section 4, including two of the direct studies of the cachectic heart, were obtained in colorectal tumor models, namely the C26 colon carcinoma and the *Apc*^Min/+^ model of intestinal tumorigenesis (Manne et al., 2013; Tian et al., 2011; Zerrouk et al., 2026). It also shows that tumor BCAA metabolism need not follow the suppressed catabolic pattern found in most malignancies (Ericksen et al., 2019): in C26 tumor-bearing mice, tumor BCAA content and BCKDH activity were higher at four weeks than at two weeks, whereas BCAA concentrations fell in skeletal muscle and kidney (Zerrouk et al., 2026). In colorectal cancer cells, cytosolic BCAT1 promoted epithelial-to-mesenchymal transition and tumor spread, whereas mitochondrial BCAT2 had the opposite effect, and dietary BCAA restriction impaired the spread of BCAT1-high tumors; the subcellular location rather than the isoform was decisive, since mitochondria-targeted BCAT1 and cytosolic BCAT2 reversed these functions, and the diet reduced peritoneal dissemination without affecting primary tumor growth (Ji et al., 2026). In the hearts of C26 tumor-bearing mice, atrophic remodeling has been documented, but myocardial BCAA handling was not assessed (Tian et al., 2011). The FOLFOX and FOLFIRI regimens used to treat colorectal cancer are an additional confounder of muscle BCAA data (Section 7).

## 6 Translational implications

In HCC, mTOR inhibitors have shown only modest antitumor activity: everolimus did not improve overall survival in a phase III trial, and its combination with sorafenib was limited by intolerable toxicity (Ashworth and Wu, 2014). Notably, pancreatic ductal adenocarcinomas, typically driven by *KRAS* (Kirsten rat sarcoma viral oncogene homolog) mutations, exhibit distinct metabolic responses to mTOR pathway inhibition compared to HCC, underscoring the importance of tumor type specific metabolic profiles. These findings indicate that therapeutic interventions aimed at the metabolic pathways of branched-chain amino acids (BCAA) directly, as opposed to focusing solely on the downstream signaling of mTOR, may yield superior tumor selectivity and more favorable therapeutic results (Sivanand and Vander Heiden, 2020). Another therapeutic option is pharmacological inhibition of BCKDK (the enzyme responsible for suppressing BCAA degradation) using compounds such as BT2 which reinstates normal BCAA catabolic flux, thereby attenuating mTORC1 signaling and obstructing neoplastic proliferation (Ericksen and Han, 2019). Quantitative tracing also indicates that suppression of BCKDK induces BCAA oxidation primarily in skeletal muscle (Neinast et al., 2019).

These tumor-directed strategies act on pathways on which host muscle also depends. Activating Akt–mTORC1 signaling reversed cancer-related muscle wasting in mice (Geremia et al., 2022), so systemic mTOR inhibition could aggravate the anabolic resistance described in Section 4.1. Likewise, relieving BCKDK-mediated inhibition of BCAA catabolism restrains tumor growth (Ericksen and Han, 2019), whereas muscle-specific deletion of *Bckdk*, which is expected to relieve the same inhibition, lowered muscle BCAA content and mTOR signaling and accelerated cachexia in mice bearing Lewis lung carcinoma (Chen et al., 2024). The net effect of such interventions on the tumor-bearing host therefore cannot be predicted from tumor data alone.

Timing also matters. Longitudinal imaging work across advanced cancers supports a time-dependent shift from muscle states that still respond to intervention toward irreversible wasting nearer the end of life. This strengthens the interpretation that BCAA-focused strategies are most plausible earlier, and only when combined with approaches that dampen inflammation, ER stress signaling, and mitochondrial dysfunction rather than as stand-alone supplementation (Compton et al., 2024; Prado et al., 2013).

## 7 Limitations and open questions

Several limitations should be considered when interpreting the evidence summarized here. The first concerns tumor heterogeneity. The direction of BCAA metabolic reprogramming is not uniform across malignancies: tissue of origin determines, even among tumors with the same driver mutation, whether a tumor increases or decreases BCAA uptake and catabolic flux (Mayers et al., 2016), and although suppression of BCAA catabolism is common (Ericksen et al., 2019), it is not universal. In breast cancer, BCAT1 and other BCAA catabolic enzymes are upregulated rather than downregulated (Zhang and Han, 2017); in gliomas, BCAT1 expression is confined to tumors with wild-type IDH1 and IDH2 (Tönjes et al., 2013); and in C26 colon carcinoma, tumor BCAA content and BCKDH activity increase as the tumor progresses (Zerrouk et al., 2026). This review uses lung cancer and PDAC as its principal examples, and conclusions drawn from these two malignancies should not be generalized to tumors with a different underlying BCAA phenotype.

A second limitation concerns cachexia stage. By international consensus, cancer cachexia is a continuum comprising precachexia, cachexia and refractory cachexia, and these stages differ in reversibility and in the appropriateness of anabolic intervention (Fearon et al., 2011). Anabolic potential is not uniformly present across this trajectory (Prado et al., 2013), and longitudinal body-composition data support a time-dependent shift toward irreversible wasting (Compton et al., 2024). Metabolic findings also depend on the time point sampled: in C26 tumor-bearing mice, tumor BCAA levels and BCKDH activity were higher at four weeks than at two weeks, and peripheral tissue wasting was minimal at the earlier time point (Zerrouk et al., 2026). Most preclinical studies report a single endpoint, and most human studies are cross-sectional and include patients at different stages. The consensus group identified the inclusion of terminal-stage and earlier-stage patients within single trials as a specific weakness of earlier cachexia intervention studies (Fearon et al., 2011), and the same caution applies to the BCAA literature summarized here.

Third, nutritional status and anticancer treatment are difficult to separate from the effect of the tumor itself. Chemotherapy alone is sufficient to induce a cachectic phenotype: in tumor-free mice, five weeks of FOLFOX or FOLFIRI caused loss of muscle mass and strength together with mitochondrial depletion, without changes in ubiquitin-dependent proteolysis (Barreto et al., 2016). Cytotoxic treatment also acts directly on the BCAA machinery, since in myotubes exposed to FOLFIRI, expression of the BCAA transporter LAT1 and of PP2Cm fell, inhibitory phosphorylation of BCKDH-E1α increased, and BCKDH activity and intracellular BCAA concentrations declined (Mora and Adegoke, 2024). The heart is affected in parallel: doxorubicin produces a dose-dependent loss of cardiac mass in mice that is dependent on MuRF1, and a decrease in cardiac mass is detectable by magnetic resonance imaging in patients within one month of anthracycline exposure (Willis et al., 2019). Because most human BCAA data are obtained from patients receiving treatment, the contributions of the tumor, of reduced intake and of therapy cannot be fully separated in the available literature.

Fourth, circulating BCAA concentrations are an imperfect proxy for tissue BCAA metabolism. Plasma levels reflect the net balance of appearance and disposal across all tissues rather than flux within any one of them, and quantitative isotopic tracing shows that BCAA oxidation is distributed across multiple organs, including skeletal muscle, brown adipose tissue, liver, kidneys and heart (Neinast et al., 2019). Dissociation between compartments has been documented. In one study of failing hearts, intramyocardial BCKA accumulated while tissue BCAA concentrations remained largely unchanged, and in plasma only one of the three BCKA species was significantly elevated (Sun et al., 2016). In C26 tumor-bearing mice, BCAA accumulated in the tumor while skeletal muscle and kidney were depleted, and LAT1 expression fell in every peripheral tissue examined (Zerrouk et al., 2026), so a single circulating value represents the sum of opposing tissue-level changes and may be further dissociated from intracellular availability by altered transporter expression. Reports of elevated circulating BCAA before a pancreatic cancer diagnosis (Mayers et al., 2014) should therefore be read as evidence of altered whole-body handling rather than of any particular tissue mechanism.

Fifth, it remains unresolved whether BCAA abnormalities are causal, compensatory, or downstream markers of systemic disease, and the answer may differ by tissue and by stage. Causal evidence exists at both ends of the tumor-host axis: loss of BCAA catabolism promotes tumor development and progression (Ericksen et al., 2019), while muscle-specific deletion of *Bckdk* in mice bearing Lewis lung carcinoma accelerated the onset of cachexia and shortened survival, and a BCAA-enriched diet improved survival in the knockout animals (Chen et al., 2024). Other observations are better read as adaptive. Suppression of BCAA catabolism in the stressed heart has been proposed to be compensatory at early stages, sparing amino acids from oxidation for protein synthesis (Sun et al., 2016); the switch of cardiac glucose transporter isoforms in C26 tumor-bearing mice may compensate for late-stage hypoglycemia (Tian et al., 2011); and the soleus maintained mTORC1 signaling despite reduced BCAA availability, indicating muscle-specific adaptation (Zerrouk et al., 2026). Others again are best interpreted as markers, since elevation of circulating BCAA years before a pancreatic cancer diagnosis (Mayers et al., 2014) establishes a temporal association but not a causal role. Notably, restoring intracellular BCAA concentrations by supplementation did not rescue atrophy or mTORC1 signaling in chemotherapy-treated myotubes, whereas maintaining LAT1 expression did (Mora and Adegoke, 2024), indicating that substrate availability is not the only limiting step. These possibilities are not mutually exclusive, and the available data do not support a single interpretation across tissues and disease stages.

Finally, the mechanistic framework applied here to the cachectic heart is largely extrapolated from heart failure and from genetic models of impaired BCAA catabolism (Huang et al., 2011; Lu et al., 2009; Sun et al., 2016), and this extrapolation remains untested. Direct measurements in tumor-bearing hosts are scarce: a study profiling BCAA and BCKA across multiple tissues in C26 tumor-bearing mice did not include the heart (Zerrouk et al., 2026); metabolite profiling in a cachexia-inducing PDAC xenograft reported reduced BCAA content in the lungs, spleen and pancreas (valine only in the pancreas) but not in the heart (Sharma et al., 2025); and a leucine-rich diet attenuated cardiac proteolytic activity and markers of cardiac injury in Walker-256 tumor-bearing rats, without measurement of myocardial BCAA catabolism (Toneto et al., 2016). In that study the loss of heart mass itself was not prevented by the diet. Model and tracer differences add further uncertainty, since isotopic tracing indicates that the major metabolic fate of branched-chain keto acids in the heart is reamination to valine rather than oxidation, attributed to low myocardial expression of the mitochondrial BCAA transporter SLC25A44 (Walejko et al., 2021), whereas quantitative tracing in other work places the heart among the tissues with appreciable BCAA oxidation (Neinast et al., 2019). The two studies followed different tracers in different species - labeled α-ketoisovalerate in rat hearts and labeled BCAA in mice - and are therefore not necessarily in conflict. The consensus framework for cancer cachexia notes explicitly that tissues other than skeletal muscle, cardiac muscle among them, should not be overlooked (Fearon et al., 2011). Until myocardial BCKDH activity, PP2Cm expression and intramyocardial BCKA content are measured directly in tumor-bearing hosts, the cardiac component of the framework presented here should be regarded as a hypothesis to be tested rather than an established mechanism.

## 8 Discussion and conclusions

The significance of BCAA lies in their involvement in bioenergetics, anabolic regulation, and adaptation to stress in striated muscle. A key theme emerging from this review is that skeletal and cardiac muscle handle BCAA through partially shared pathways but under distinct physiological constraints. Skeletal muscle is a dominant site of whole-body BCAA transamination and contributes substantially to systemic nitrogen trafficking, while the myocardium maintains relatively low baseline BCAA oxidation and relies primarily on fatty acid and glucose metabolism to meet a continuous energetic demand. This tissue specificity is underpinned by differential regulation of the BCAT and BCKDH axis and its modulators, BCKDK and PP2Cm, and it becomes consequential under systemic disease stress.

Cancer reprograms BCAA metabolism in a tumor-type-dependent manner and thereby reshapes nutrient partitioning between tumor and host (**Figure 2**). Across malignancies, suppression or rerouting of BCAA catabolic flux can sustain intracellular BCAA pools and mTORC1 signaling, supporting proliferation. The clinical relevance of this tumor-centric rewiring lies in its coupling to cachexia, in which inflammatory and endocrine disturbances enforce a net catabolic state in peripheral tissues. Cachexia should therefore not be read as a simple consequence of reduced intake, but as a biologically driven, multi-organ syndrome in which inflammatory mediators and cellular stress programs constrain anabolic responsiveness even when substrates are available.

The two malignancies used as principal examples show how little of this framework is tumor-general. They differ at the level of the tumor itself: non-small cell lung cancer takes up circulating BCAA avidly, whereas PDAC relies more on alternative nitrogen sources (Mayers et al., 2016). They differ again in which host mechanisms have actually been demonstrated. The causal evidence in skeletal muscle comes from a lung cancer model (Chen et al., 2024), whereas the causal evidence for adipose–muscle crosstalk and for the myocardium comes from pancreatic models (Rupert et al., 2021; Stelter et al., 2024). Neither set of findings has been reproduced in the other tumor type. The convergence depicted in **Figure 2** is therefore a convergence of outcomes rather than of demonstrated mechanisms, and it should be read as such.

Three unresolved points deserve emphasis. First, the direction in which BCAA catabolic flux relates to outcome is not the same in tumor and in host muscle: relieving BCKDK-mediated inhibition restrains tumor growth (Ericksen and Han, 2019), whereas muscle-specific deletion of *Bckdk* accelerated cachexia and shortened survival in tumor-bearing mice (Chen et al., 2024). A systemic intervention at this control point would therefore act in opposite directions in the two compartments. Second, the cardiac component of the framework rests on extrapolation: to our knowledge, myocardial BCKDH activity, PP2Cm expression and intramyocardial BCKA content have not been measured in a tumor-bearing host, and the two studies that profiled BCAA across tissues in tumor-bearing animals either omitted the heart or found no reduction there (Sharma et al., 2025; Zerrouk et al., 2026). Third, the normal fate of BCKA in the heart has been addressed with different tracers and species, and the results are not directly comparable, with isotopic tracing pointing to reamination in one study and to appreciable oxidation in another (Neinast et al., 2019; Walejko et al., 2021). These points are developed in Section 7; taken together, they set the limits within which the present framework can be applied.

Three inferences follow. First, BCAA metabolism cannot be interpreted uniformly across striated tissues, because skeletal and cardiac muscle differ in their baseline reliance on BCAA oxidation and in their vulnerability to the accumulation of catabolic intermediates. Second, tumor-driven BCAA reprogramming interacts with inflammation, mitochondrial dysfunction and impaired anabolic signaling to produce a state in which muscle loss is driven by these processes rather than by substrate limitation alone. Third, BCAA-focused strategies are unlikely to be effective as stand-alone measures in established cachexia, both because anabolic potential declines along the disease trajectory (Compton et al., 2024; Prado et al., 2013) and because restoring BCAA availability does not by itself overcome impaired amino acid transport and anabolic signaling (Mora and Adegoke, 2024). Future work should therefore prioritize tissue-specific markers of BCAA flux and catabolic control, define the window in which anabolic potential remains exploitable, and test combined interventions that preserve muscle and cardiac function without compromising oncologic efficacy. What this review contributes is a tissue-specific reading of the existing evidence, with the untested parts identified as such; it is not a therapeutic model ready for clinical use.

## Glossary

Akt: protein kinase B
ATP: adenosine triphosphate
BCAA: branched-chain amino acid
BCAT: branched-chain aminotransferase
BCAT1: cytosolic branched-chain aminotransferase (BCATc)
BCAT2: mitochondrial branched-chain aminotransferase (BCATm)
BCKA: branched-chain α-keto acid
BCKDH: branched-chain α-keto acid dehydrogenase
BCKDHA: E1α subunit of the BCKDH complex
BCKDK: branched-chain α-keto acid dehydrogenase kinase
BT2: small-molecule inhibitor of BCKDK
C26: colon-26 carcinoma
CCL2: C-C motif chemokine ligand 2
CCR2: C-C motif chemokine receptor 2
CoA: coenzyme A
CPT1β: carnitine palmitoyltransferase 1β
E1/E2/E3: catalytic components of the BCKDH complex
ER: endoplasmic reticulum
FAD/FADH2: flavin adenine dinucleotide (oxidized/reduced)
FOLFIRI: folinic acid, fluorouracil and irinotecan
FOLFOX: folinic acid, fluorouracil and oxaliplatin
GTP: guanosine triphosphate
HCC: hepatocellular carcinoma
IDH1/IDH2: isocitrate dehydrogenase 1 and 2
IFN-γ: interferon-γ
IL-1β: interleukin-1β
IL-6: interleukin-6
IRE1α: inositol-requiring enzyme 1α
JAK: Janus kinase
KIC: α-ketoisocaproate
KIV: α-ketoisovalerate
KLF15: Krüppel-like factor 15
KMV: α-keto-β-methylvalerate
KPC: genetically engineered mouse model of pancreatic carcinoma
KRAS: Kirsten rat sarcoma viral oncogene homolog
LAT1: large neutral amino acid transporter 1
LVEF: left ventricular ejection fraction
MAO-A: monoamine oxidase A
mTOR: mechanistic target of rapamycin
mTORC1: mechanistic target of rapamycin complex 1
MuRF1: muscle RING-finger protein 1
NAD+/NADH: nicotinamide adenine dinucleotide (oxidized/reduced)
NF-κB: nuclear factor κB
NSCLC: non-small cell lung cancer
PAAD: pancreatic adenocarcinoma (TCGA cohort designation)
PDAC: pancreatic ductal adenocarcinoma
PI3K: phosphoinositide 3-kinase
PLP: pyridoxal 5′-phosphate
PP2Cm: mitochondrial protein phosphatase 2C
PPARα: peroxisome proliferator-activated receptor α
PPM1K: gene encoding PP2Cm
ROS: reactive oxygen species
sIL-6R: soluble interleukin-6 receptor
SLC25A44: solute carrier family 25 member 44
STAT3: signal transducer and activator of transcription 3
TCA: tricarboxylic acid
TNF-α: tumor necrosis factor-α
TPP: thiamine pyrophosphate
TSC1/TSC2: tuberous sclerosis complex 1 and 2
TWEAK: TNF-related weak inducer of apoptosis
UPR: unfolded protein response
UPS: ubiquitin–proteasome system
XBP1: X-box binding protein 1
